# Status and influencing factors of balance in middle-aged and older adults with Parkinson’s disease: a national longitudinal study

**DOI:** 10.3389/fneur.2025.1499640

**Published:** 2025-04-30

**Authors:** Wu-xiao Wei, Xin-gui Zhuo, Hong-qiao Chen, Ming-li Chen

**Affiliations:** ^1^Guangxi University of Science and Technology First Affiliated Hospital, Liuzhou, China; ^2^Wuzhou Medical College, Wuzhou, China

**Keywords:** Parkinson’s disease, balance, CHARLS, middle-aged and older patients, national longitudinal study

## Abstract

**Background:**

To examine the current status and influencing factors of balance in middle-aged and older adults with Parkinson’s disease (PD) and explore the correlations of these factors with balance.

**Methods:**

The China Health and Retirement Longitudinal Study (CHARLS) Database in 2015, 2018 and 2020 were utilized as the data source, from which the missing value samples were excluded and 1,390 participants aged ≥45 years were recruited. Using the chi-square test, balance comparisons were made among middle-aged and older adults PD patients under different indicators. The influences of different factors on the patient balance were investigated through regression analysis.

**Results:**

Regression analysis revealed the correlations of age, gender, smoking, falls, hypertension, diabetes and physical activity with balance in PD patients. A significant association between aging and declined balance was found, with middle-aged and older adults PD patients aged > 65 years showing a higher probability of declined balance (OR = 0.716, *p* = 0.016). Male middle-aged and older adults PD patients exhibited better balance than female counterparts (OR = 1.829, *p* = 0.001). Previous smoking (OR = 0.580, *p* = 0.004), falls (OR = 0.769, *p* = 0.035), hypertension (OR = 0.738, *p* = 0.019) and diabetes (OR = 0.734, *p* = 0.027) were positively correlated with the declined balance in PD patients. Light physical activity could significantly improve balance in middle-aged and older adults PD patients (OR = 1.672, *p* < 0.001).

**Conclusion:**

Balance impairment is a major concern for middle-aged and older adults with PD. Our findings highlight that age, gender, smoking, history of falls, hypertension, diabetes, and physical activity significantly influence balance. Specifically, old age, male gender, light physical activity (such as walking), and lower risks of hypertension and diabetes are linked to better balance. Clinicians should focus on managing these risk factors and promoting light physical activity to improve balance and reduce fall risks.

## 1 Introduction

Parkinson’s disease (PD) is the second most common neurodegenerative disease after Alzheimer’s disease (1, 2). PD is rare in individuals under 50, and its prevalence increases with age (3). It is projected that the number of cases will reach between 8.7 million and 9.3 million by 2030 (3, 4). Main clinical features of PD include muscle rigidity, static tremor, bradykinesia, postural instability and gait disorders (5). Postural instability accompanied by increased postural swing and impaired response to external disturbances may lead to impaired balance, and with the disease progression, patients may lose their self-care ability and their physical and mental health may be affected (5, 6).

Among motor and non-motor symptoms of PD, the gait and posture symptoms deteriorate at faster rates, and the muscle and core control functions in PD patients gradually become impaired due to declined balance and postural reflex (7–9). Balance is a complex body function of multi-system coordination (10). Impaired balance is the principal cause of falls (11) among PD patients, resulting in injury, fracture and even disability, which will severely limit their ability to perform activities of daily living, and negatively affect their quality of life and self-care confidence (12, 13).

Balance dysfunction in PD patients leads to fall injuries, fractures and even disability, which are important reasons for increased healthcare costs (14–16). Over the past 20 years, the total number of PD patients has reached 3.62 million (in 2020) in China, which will continue to rise in the future, resulting in further increased burden from PD in the next few decades with the population aging (17–19). Thoroughly understanding the status quo and influencing factors of PD has become a major health issue of global concern, which is critical to intervening in the factors affecting declined balance, ameliorating the balance and improving the functional capacity of PD patients (20).

Given the increasing prevalence of PD and its associated motor symptoms, particularly balance dysfunction, it is crucial to understand the factors influencing balance in middle-aged and older PD patients. Although existing research has emphasized various aspects of balance in PD, little is known about how specific risk factors, such as age, gender, smoking history, comorbidities (such as hypertension and diabetes), and physical activity (such as walking), affect balance in this population. Therefore, based on the 2015, 2018, and 2020 China Health and Retirement Longitudinal Study (CHARLS) database, we aim to provide evidence on the specific impacts of these factors and identify potential intervention strategies to help improve balance and reduce the risk of falls in PD patients.

## 2 Methods

### 2.1 Study population

The data for this study was obtained from CHARLS (21) using datasets from 2015, 2018, and 2020. The sampling method employed was probability proportional to size (PPS), where 150 county-level units were randomly selected from all units (excluding Tibet, Taiwan, Hong Kong, Macau, Ningxia, and Hainan). These were further categorized by region, urban/rural status, and GDP, with the lowest level of government organizations (including administrative villages) as primary sampling units. A total of 450 communities/villages were included, though four lacked data in 2020.

**Figure 1:**
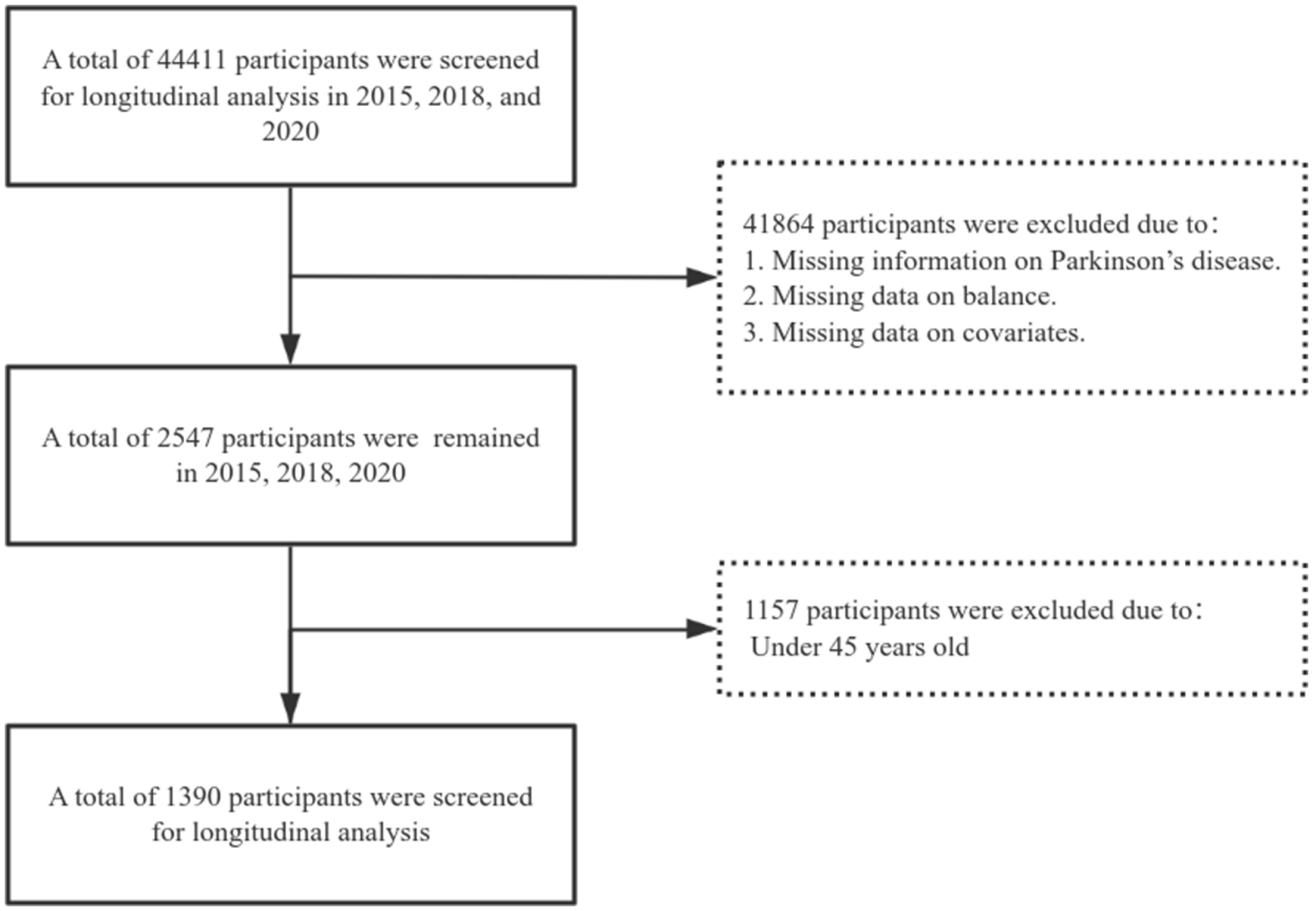
Flowchart showing the selection of participants into the final analysis of this study.

To ensure data quality and consistency, participants were excluded based on the following criteria: (1) participants missing Parkinson’s disease information, (2) participants missing balance data, (3) participants missing covariate information, and (4) participants under the age of 45. In total, 1,390 eligible participants were included in the analysis. The detailed sample selection process is shown in **Figure** **1**.

### 2.2 Diagnosis of PD

The PD diagnosis was based on previous diagnosis by a doctor. Participants were asked whether they had received an official PD diagnosis. If so, corresponding participants were considered to have PD; if not so, corresponding participants were considered not to have PD.

### 2.3 Balance assessment

Interviewers were responsible for measuring balance, which refers to the ability of participants to stand in the semi-tandem position for 10 s without moving or holding anything.

### 2.4 Covariates

Covariates included Age, Gender, Residence, Educational level, Marital status, Smoking, Drinking, Falls, Light (Include walking, which involves moving from one place to another while working or at home, as well as other walks you take for leisure, exercise, training, or entertainment.), Moderate (Moderate physical activities make your breathing faster than usual, such as carrying light items, cycling at a regular pace, mopping, practicing tai chi, brisk walking, and so on.) and Heavy physical activities (Intense activities will make you breathe heavily, such as carrying heavy objects, digging, farming, aerobic exercises, cycling quickly, and biking with cargo.), Depression, Disability, Physical pain, Hypertension, Diabetes, Dyslipidemia, Heart disease, Stroke and Cancer. Depressive symptoms were measured by the 10-item version of CES-D (CESD-10), which has been validated and widely used in Chinese adults (22–24). It consists of 10 items: (1) feeling depressed, (2) being bothered by small things, (3) difficulty concentrating, (4) restless sleep, (5) everything is effort, (6) feeling hopeful, (7) feeling happy, (8) feeling fearful, (9) unable to get going, and (10) feeling lonely. Each item was scored on a four-point Likert scale comprising “Rarely or none of the time,” “Some or a little of the time,” “Occasionally or a moderate amount of time” and “Most or all of the time,” with negative symptoms designated as 0, 1, 2 and 3. Contrastively, two positive symptoms were designated as 3, 2, 1, and 0. The scale has a total score between 0 and 30, with higher scores indicating more depressive symptoms. A cutoff score of ≥10 was utilized to identify respondents exhibiting significant depressive symptoms (22).

### 2.5 Statistical analysis

The factors influencing the balance of middle-aged and older adults PD patients were analyzed by establishing Logistics model and calculating OR values. The basic model can be written as follows:


$$\begin{array}{l} \mathrm{Balanc}e_{\mathrm{it}}=\beta_{0}+\beta_{1}Age_{\mathrm{it}}+\beta_{2}\mathrm{Gende}r_{\mathrm{it}}+\beta_{3}{BMI}_{\mathrm{it}}+\beta_{4}{\mathrm{Residence}}_{\mathrm{it}}+\\ \beta_{5}{\mathrm{Educational level}}_{\mathrm{it}}+\beta_{6}{\mathrm{Marital status}}_{\mathrm{it}}+\\ \beta_{7}{\mathrm{Smoking}}_{\mathrm{it}}+\beta_{8}\mathrm{Drinkin}g_{\mathrm{it}}+\beta_{9}\mathrm{Fall}s_{\mathrm{it}}+\\ \beta_{10}\mathrm{Heavy}\;{\mathrm{physical activities}}_{\mathrm{it}}+\\ \beta_{11}\mathrm{Moderate}\;{\mathrm{physical activities}}_{\mathrm{it}}+\\ \beta_{12}\mathrm{Light}\;{\mathrm{physical activities}}_{\mathrm{it}}+\beta_{13}{\mathrm{Depression}}_{\mathrm{it}}+\\ \beta_{14}\mathrm{Disa}b\mathrm{ilit}y_{\mathrm{it}}+\beta_{15}{\mathrm{Physical pain}}_{\mathrm{it}}+\\ \beta_{16}\mathrm{Hypertensio}n_{\mathrm{it}}+\beta_{17}{\mathrm{Diabetes}}_{\mathrm{it}}+\\ \beta_{18}{\mathrm{Dyslipidemia}}_{\mathrm{it}}+\beta_{19}{\mathrm{Heart disease}}_{\mathrm{it}}+\\ \beta_{20}{\mathrm{Stroke}}_{\mathrm{it}}+\beta_{20}\mathrm{Cance}r_{\mathrm{it}}+\varepsilon_{\mathrm{it}} \end{array}$$

where i represents different individuals, t represents years (2015/2018 and 2020), *β*_0_ stands for the intercept term, and $\varepsilon_{\mathrm{it}}$ stands for the random error term.Descriptive statistics were first conducted to summarize the demographic and clinical characteristics of the participants. For categorical variables, the chi-square test was applied to compare the balance status (good vs. poor).To assess the relationships between the various factors and balance in middle-aged and older adults PD patients, we used logistic regression analysis. This analysis was performed to calculate the odds ratios (ORs). All statistical analyses were performed with STATA 16.0 software, with a significance level set at 0.05.

## 3 Result

### 3.1 Difference analysis

Based on the results from Table 1, patients aged 65 years and above were more likely to experience poor balance compared to those aged 45–65 years, with a statistically significant difference (χ^2^ = 20.901, *p* < 0.001).Male patients exhibited better balance than female patients, and this difference was significant (χ^2^ = 7.342, *p* = 0.007).Married patients had better balance than those who were divorced, widowed, or unmarried, with a significant difference (χ^2^ = 12.313, *p* < 0.001).Current smokers had better balance compared to past smokers and never smokers, with a statistically significant difference (χ^2^ = 12.711, *p* = 0.002).Patients with a history of falls had worse balance compared to those without a fall history, and this difference was statistically significant (χ^2^ = 7.498, *p* = 0.006).Patients engaged in light physical activity showed significantly better balance compared to those who did not (χ^2^ = 38.810, *p* < 0.001). Similarly, patients involved in moderate and heavy physical activities also had better balance than those not engaged in such activities (*p* < 0.05 for both).Patients with hypertension and diabetes were more likely to have poorer balance, with significant differences observed (Hypertension: χ^2^ = 11.985, *p* = 0.001; Diabetes: χ^2^ = 6.969, *p* = 0.008).

**Table 1:** Balance comparisons of middle-aged and older adults PD patients under different indicators.

Category	Subcategory	Good balance		Poor balance		χ2	*p-*value
		Sample number	Proportion (%)	Sample number	Proportion (%)		
Age	45–65 years	311	36.04	128	24.29	20.901	<0.001
	≥65 years	552	63.96	399	75.71		
Gender	Female	430	49.83	302	57.31	7.342	0.007
	Male	433	50.17	225	42.69		
Residence	Rural	542	62.8	330	62.62	0.005	0.945
	Urban	321	37.2	197	37.38		
Educational level	Primary school and below	603	69.87	389	73.81	2.594	0.273
	Junior high school	169	19.58	92	17.46		
	Senior high school and above	91	10.54	46	8.73		
Marital status	Others	169	19.58	146	27.7	12.313	<0.001
	Married	694	80.42	381	72.3		
Smoking	Never	493	57.13	309	58.63	12.711	0.002
	Past smoking	158	18.31	126	23.91		
	Current smoking	212	24.57	92	17.46		
Drinking	Never	491	56.89	321	60.91	7.297	0.026
	Past drinking	108	12.51	79	14.99		
	Current drinking	264	30.59	127	24.1		
Falls	No	580	67.21	316	59.96	7.498	0.006
	Yes	283	32.79	211	40.04		
Heavy physical activities	No	645	74.74	459	87.1	30.575	<0.001
	Yes	218	25.26	68	12.9		
Moderate physical activities	No	513	59.44	394	74.76	33.864	<0.001
	Yes	350	40.56	133	25.24		
Light physical activities	No	219	25.38	218	41.37	38.810	<0.001
	Yes	644	74.62	309	58.63		
Depression	No	439	50.87	318	60.34	11.838	0.001
	Yes	424	49.13	209	39.66		
Disability	No	256	29.66	116	22.01	9.776	0.002
	Yes	607	70.34	411	77.99		
Physical pain	No	224	25.96	154	29.22	1.763	0.184
	Yes	639	74.04	373	70.78		
Hypertension	No	362	41.95	172	32.64	11.985	0.001
	Yes	501	58.05	355	67.36		
Diabetes	No	658	76.25	368	69.83	6.969	0.008
	Yes	205	23.75	159	30.17		
Dyslipidemia	No	472	54.69	290	55.03	0.015	0.903
	Yes	391	45.31	237	44.97		
Heart disease	No	449	52.03	293	55.6	1.676	0.196
	Yes	414	47.97	234	44.4		
Stroke	No	646	74.86	368	69.83	4.188	0.041
	Yes	217	25.14	159	30.17		
Cancer	No	835	96.76	513	97.34	0.386	0.534
	Yes	28	3.24	14	2.66		

PD: Parkinson’s disease; *χ^2^* represents the Chi-square test. *p*-values < 0.05 are considered statistically significant.

### 3.2 Correlation coefficient analysis

A correlation analysis was conducted based on the results from Table 2 to examine the relationship between balance ability and various explanatory variables in middle-aged and older adults Parkinson’s disease patients. A significant negative correlation was found between age and balance ability (*r* = −0.123, *p* < 0.01). Gender showed a small but significant positive correlation with balance ability (*r* = 0.073, *p* < 0.01). Smoking was significantly positively correlated with balance ability (*r* = 0.051, *p* < 0.05). Various forms of physical activity (light, moderate, and vigorous) were positively correlated with balance ability, with the strongest correlation observed for light physical activity (*r* = 0.167, *p* < 0.01), followed by moderate physical activity (*r* = 0.156, *p* < 0.01) and vigorous physical activity (*r* = 0.148, *p* < 0.01). This suggests that participation in physical activity can improve the balance ability of Parkinson’s disease patients.

**Table 2:** Correlation coefficient analysis.

	Balance	Age	Gender	Smoking	Hypertension	Diabetes	HPA	MPA	LPA
Balance	1								
Age	−0.123	1							
Gender	0.073	0.077	1						
Smoking	0.051	−0.002	0.610	1					
Hypertension	−0.093***	0.071***	−0.004	0.092***	1				
Diabetes	−0.071***	0.017	−0.076***	0.063**	0.065**	1			
HPA	0.148***	−0.183***	0.052*	0.01	−0.164***	−0.014	1		
MPA	0.156***	−0.190***	−0.065**	0.023	−0.069**	0.021	0.077***	1	
LPA	0.167***	−0.107***	−0.019	0.014	0.048*	0.071***	0.089***	−0.007	1

*, **, *** represent significance levels of 10, 5, and 1%, respectively. HPA: Heavy physical activities. MPA: Moderate physical activities. LPA: Light physical activities.

### 3.3 Baseline regression analysis

Based on the results from Tables 3, a baseline logistic regression analysis was conducted to explore the factors influencing balance in middle-aged and older adults PD patients. Patients aged 65 years and above have a significantly higher likelihood of poor balance compared to those aged 45–65 years (OR = 0.716, *p* = 0.016), indicating that old age is associated with worse balance. Male patients are significantly more likely to have better balance than female patients (OR = 1.829, *p* = 0.001), suggesting a positive correlation between male gender and balance. Past smokers are associated with a significantly higher probability of poor balance compared to those who never smoked (OR = 0.580, *p* = 0.004), indicating that a history of smoking negatively affects balance in PD patients. Patients with a history of falls are more likely to experience poor balance compared to those without a history of falls (OR = 0.769, *p* = 0.035), confirming that falls have a detrimental effect on balance. Light physical activity is significantly associated with better balance (OR = 1.672, *p* < 0.001). Additionally, both moderate (OR = 1.358, *p* = 0.029) and heavy (OR = 1.550, *p* = 0.010) physical activities also improve balance, indicating that physical activity, in general, promotes better balance in PD patients. Both hypertension (OR = 0.738, *p* = 0.019) and diabetes (OR = 0.734, *p* = 0.027) are significantly associated with a higher likelihood of poor balance, suggesting that these conditions negatively impact balance in PD patients. In summary, old age, past smoking, a history of falls, hypertension, and diabetes are significant risk factors for poor balance in PD patients. Conversely, being male and engaging in physical activity, especially light activity, are associated with better balance.

**Table 3:** Baseline regression results.

Variable	Category	Coefficient	SD	*χ* ^2^	*P*	OR
Age	45–65 years	–	–	–	–	1
	≥65 years	−0.333	0.139	−2.400	0.016	0.716
Gender	Female	–	–	–	–	1
	Male	0.604	0.177	3.420	0.001	1.829
BMI	BMI	−0.011	0.008	−1.350	0.177	0.989
Residence	Rural	–	–	–	–	1
	Urban	0.054	0.130	0.420	0.677	1.056
Educational level	Primary school and below	–	–	–	–	1
	Junior high school	−0.178	0.163	−1.090	0.275	0.837
	Senior high school and above	−0.033	0.216	−0.150	0.880	0.968
Marital status	Others	–	–	–	–	1
	Married	0.161	0.143	1.130	0.259	1.175
Smoking	Never	–	–	–	–	1
	Past smoking	−0.545	0.188	−2.890	0.004	0.580
	Current smoking	−0.040	0.189	−0.210	0.832	0.961
Drinking	Never	–	–	–	–	1
	Past drinking	−0.044	0.184	−0.240	0.812	0.957
	Current drinking	0.058	0.153	0.380	0.704	1.060
Falls	No	–	–	–	–	1
	Yes	−0.262	0.124	−2.110	0.035	0.769
Heavy physical activities	No	–	–	–	–	1
	Yes	0.438	0.170	2.570	0.010	1.550
Moderate physical activities	No	–	–	–	–	1
	Yes	0.306	0.140	2.180	0.029	1.358
Light physical activities	No	–	–	–	–	1
	Yes	0.514	0.130	3.950	<0.001	1.672
Depression	No	–	–	–	–	1
	Yes	0.218	0.124	1.760	0.079	1.243
Disability	No	–	–	–	–	1
	Yes	−0.268	0.139	−1.920	0.055	0.765
Physical pain	No	–	–	–	–	1
	Yes	0.108	0.139	0.780	0.437	1.114
Hypertension	No	–	–	–	–	1
	Yes	−0.303	0.130	−2.340	0.019	0.738
Diabetes	No	–	–	–	–	1
	Yes	−0.309	0.140	−2.210	0.027	0.734
Dyslipidemia	No	–	–	–	–	1
	Yes	0.110	0.135	0.820	0.413	1.117
Heart disease	No	–	–	–	–	1
	Yes	0.281	0.126	2.220	0.026	1.324
Stroke	No	–	–	–	–	1
	Yes	−0.154	0.138	−1.120	0.262	0.857
Cancer	No	–	–	–	–	1
	Yes	0.379	0.351	1.080	0.280	1.461

BMI: body mass index. SD is the standard deviation of the coefficient.χ^2^ is the chi-squared statistic for testing the significance of the variable. P is the *p*-value for the hypothesis test, indicating the statistical significance of each variable. OR indicates the odds of the outcome occurring for each category relative to the reference category.

## 4 Discussion

The results indicate that several factors significantly influence balance in middle-aged and older adults with PD. These include age, gender, smoking history, history of falls, hypertension, diabetes, and physical activity levels. Old age, male gender, a history of smoking, prior falls, and the presence of hypertension and diabetes are associated with worse balance. Conversely, engaging in light physical activity, such as walking, is positively correlated with better balance. The findings suggest that balance dysfunction in PD is influenced by multiple risk factors, and that managing these factors, especially encouraging physical activity, may help improve balance and reduce fall risk in this population.

Balance disorder, as a common dysfunction of PD, is associated with abnormalities in the nigrostriatal and vestibular neural pathways (15, 25). Early clinical detection of balance abnormalities and assessment of falls are conducive to the comprehensive management of PD patients. Through measurement with dynamic balance instrument, Jong Moon Lee et al. (26) found that patients with early PD had accompanying balance disorder, which might be associated with age and disease course. PD is accompanied by a history of falls, which tend to increase gradually with the disease progression, resulting in post-fall injuries such as fractures, limited activities and psychological disorders (27). Successful standing on one leg for 10 s is conducive to predicting survival in middle-aged and older adults (28).

Fall events in PD are the result of interactions among multiple factors, of which balance disorder is regarded as the major cause, as well as an important factor for the limited activities of daily living and social participation in PD patients (8, 9, 11, 29). Research has shown that the dynamic and static center of mass (COM) trajectories and motion speeds in PD patients were prominently prolonged and accelerated, while the limits of stability were reduced, indicating balance dysfunction in the early- and middle-stage PD patients, which further affected their postural control and gait stability (30, 31). In particular, PD patients often face impaired balance and compromised motor coordination with age, which can lead to postural instability, difficulty walking and higher probability of falls (6, 8, 11, 25, 32, 33). The study by Murueta-Goyena et al. suggests that female patients are at a higher risk of falling due to issues with motor fluctuations and postural stability. This may explain why middle-aged and old male Parkinson’s disease patients tend to exhibit better balance than their female counterparts (34). Some studies have demonstrated an association between long-term smoking and a lower risk of PD, with the duration and intensity of smoking being more important, while the number of cigarettes smoked per day is generally irrelevant to the risk of PD (35–38). Although smoking may be associated with a lower risk of PD in some cases, it can interfere with neurological function and adversely affect balance control in middle-aged and older PD patients (37). This study found worse balance dysfunction in the fall group than in the non-fall group, showing consistency with the conclusion of Bekkers (6), who found that compared to the control group, PD patients with freezing of gait might have difficulty in sensing the speed difference in motor band. When the human body is about to fall due to smaller and slower disturbances, it mainly relies on the ankle joint control to restore postural stability (ankle strategy). A decreased sense of proprioception at ankle joint affects the implementation of ankle strategy, which may also be the reason why patients are more prone to fall. Currently, the association between the hypertension and the risk of PD remains controversial (6). Hypertensive patients are more likely to develop PD compared to non-hypertensive participants (39, 40). The prevalence of peripheral neuropathy in Parkinson’s disease patients ranges from 4.8 to 55%, which is significantly higher than that in the general population. When diabetic neuropathy coexists with Parkinson’s disease, the pathological mechanisms of both conditions may overlap, further worsening balance function (41).

Many studies have shown that rehabilitation training has a preferable effect on improving postural imbalance in PD patients and reducing the occurrence of falls (42–46). M. Rossi et al. (47) found that vestibular rehabilitation training under the movement and visual changes of dynamic postural balance platform could improve the postural imbalance of PD patients to the greatest extent and reduce their risk of falls (48, 49). This study also found that light physical activities, such as walking, may help reduce the risk of balance dysfunction in Parkinson’s disease patients, providing an important foundation for improving their quality of life. This is consistent with the research by Nascimento et al. (50), which showed that walking training, especially when combined with external cues, can improve gait fluency and reduce gait freezing. It offers a low-cost, low-risk intervention for the overall health, motor function, and non-motor symptoms of Parkinson’s disease patients, with significant benefits (51).In clinical practice, attention should be paid to old patients who smoke, falls, lack physical activities and have hypertension or diabetes. For these patients, corresponding interventions should be given to improve their quality of life.

While our study provides valuable insights into the relationship between balance ability and various health factors in Parkinson’s disease, several limitations should be considered. First, the CHARLS dataset lacks detailed information on medication status, which could affect balance, particularly in populations with conditions like Parkinson’s disease. This limits our ability to analyze how medication use or timing might impact balance. Second, the dataset does not categorize Parkinson’s disease patients by clinical subtypes, which restricts our ability to examine differences in balance problems across subtypes, such as tremor-dominant or postural instability and gait failure types. Additionally, the use of self-reported PD diagnosis, rather than clinical verification, could introduce selection bias, as individuals may misreport or have inaccuracies in their diagnosis. Although we controlled for potential confounders such as age, gender, and physical activity, other unmeasured factors, such as socioeconomic factors and pre-existing conditions, may also influence balance and physical activity levels. Furthermore, the relationship between smoking and balance may be influenced by other lifestyle factors, such as diet or weight differences. Finally, the use of a two-point scale for balance assessment may not fully capture the degree of balance dysfunction. Future research could employ more detailed assessment methods to better understand balance problems.

## 5 Conclusion

Balance impairment is a significant issue for middle-aged and older adults with PD. Our results indicate that age, gender, smoking, history of falls, hypertension, diabetes, and physical activity are important factors influencing balance in PD patients. Specifically, old age, male gender, light physical activity, and lower risks of hypertension and diabetes are associated with better balance. We recommend that clinicians focus on managing these risk factors, particularly for older PD patients, and emphasize interventions that promote light physical activity (such as walking) to improve balance. Addressing these factors can significantly enhance the quality of life and reduce the risks associated with falls and postural instability in this population. Future research should explore targeted interventions and the mechanisms underlying these associations to better guide clinical practice.

## Data Availability

The datasets presented in this study can be found in online repositories. The names of the repository/repositories and accession number(s) can be found below: the data used in this study are publicly released data by CHARLS. Permissions were obtained to access the data used in our research, which were granted by the CHARLS team. The raw data is available on the website (https://charls.pku.edu.cn/en).
